# Effects of Functional Yogurt Supplementation on Aflatoxin-Induced Maternal–Fetal Toxicity in Pregnant Rats

**DOI:** 10.3390/toxins18100457

**Published:** 2026-10-20

**Authors:** João Victor Batista da Silva, Jéssica Cristina Leme, Giovana Perez Montenegro, Deisy Mara Silva Longo, Marlei Josiele Augusto, Fernando Silva Ramalho, Lucas Gabriel Dionisio Freire, Carlos Humberto Corassin, Fernando Gustavo Tonin, Sher Ali, Carlos Augusto Fernandes de Oliveira, Leandra Náira Zambelli Ramalho

**Affiliations:** 1 Department of Pathology and Forensic Medicine, School of Medicine of Ribeirão Preto, University of São Paulo, Ribeirao Preto 14049-900, Sao Paulo, Brazil; joaovsilva@usp.br (J.V.B.d.S.); jessicaleme@usp.br (J.C.L.); giovana.montenegro@usp.br (G.P.M.); dms@fmrp.usp.br (D.M.S.L.); maugusto@fmrp.usp.br (M.J.A.); framalho@fmrp.usp.br (F.S.R.); 2 Department of Food Engineering, School of Animal Science and Food Engineering, University of São Paulo, Pirassununga 13635-900, Sao Paulo, Brazil; lucasgdfreire@usp.br (L.G.D.F.); carloscorassin@usp.br (C.H.C.); alisher@usp.br (S.A.); 3 Department of Biosystem Engineering, School of Animal Science and Food Engineering, University of São Paulo, Pirassununga 13635-900, Sao Paulo, Brazil; fgtonin@usp.br

**Keywords:** aflatoxins, maternal–fetal toxicity, fetal development, biosorption, functional yogurt, *Lacticaseibacillus rhamnosus*, *Saccharomyces cerevisiae*

## Abstract

Aflatoxin (AF) exposure during pregnancy may compromise maternal health and fetal development. This study aimed to evaluate the effects of gestational exposure to AF in pregnant Wistar rats and the mitigating effect of a functional yogurt (FY) containing inactivated biomass of *Lacticaseibacillus rhamnosus* and *Saccharomyces cerevisiae*. Animals were exposed to total AF (sum AFB_1_, AFB_2_, AFG_1_ and AFG_2_: 1.0 mg/kg/day) from gestational day (GD) 6 to 15, with or without FY supplementation. Maternal and reproductive parameters, fetal development, cranial ossification and placental growth factor (PlGF) immunostaining were evaluated, along with determination of AF metabolites in maternal and fetal samples. AF exposure reduced maternal weight gain during GD6–GD15 and fetal weight at GD20. Delayed cranial ossification occurred in 51.4 and 15.6% of fetuses in AF and AF + FY groups, respectively, with most severe alterations occurring in the AF group. Serum aflatoxin M_1_ (AFM_1_) concentrations were lower (*p* = 0.002) in AF + FY than in AF groups. AF metabolites were quantified less frequently in maternal liver from FY-supplemented animals (*p* = 0.015), while placentas or fetal livers from the AF + FY group had no quantifiable levels of AF metabolites. PlGF immunostaining was highest in AF group and lowest in the FY group (*p* = 0.0007). In conclusion, FY containing *L. rhamnosus* and *S cerevisiae* biomasses attenuated some AF-induced effects on fetal development, particularly delayed cranial ossification.

## 1. Introduction

Adverse stimuli during the critical period of embryofetal development can cause irreversible damage with lifelong consequences for health [1]. Food contaminants are among these harmful stimuli, and mycotoxins such as aflatoxins (AFs) are particularly relevant in this context. These compounds are toxic secondary metabolites produced by certain fungal species that occur as contaminants in a variety of foods, particularly grains and oilseeds [2]. Several AFs occur naturally in foods, with AFB_1_, AFG_1_, AFB_2_, and AFG_2_ being the most important because of their toxic potential. In food matrices, the sum of AFB_1_, AFG_1_, AFB_2_, and AFG_2_ is known as total AF. Among them, AFB_1_ is considered the most toxic and is classified by the IARC as a Group 1 carcinogen [2,3]. In addition to the carcinogenic properties, AFB_1_ is associated with adverse effects at the maternal–fetal interface, as widely reported in the literature [4]. After ingestion, AFB_1_ is mainly bioactivated in the liver by cytochrome P450 enzymes to the reactive AFB_1_-8,9-epoxide, while biotransformation also produces AFM_1_, AFP_1_ and AFQ_1_, among other metabolites [5]. Major biomarkers of AFB_1_ exposure include AFB_1_-lysine/albumin adducts in serum [6,7] and AFM_1_ and AFB_1_-N^7^-guanine in urine [6]. AFB_1_ can also cross the placental barrier, resulting in direct fetal exposure [8]. Experimental studies in animals indicate that exposure to these compounds during embryofetal development is associated with fewer fetuses per litter, reduced fetal size, and malformations, particularly skeletal abnormalities [9,10,11,12]. In humans, the available data indicate that AF exposure during pregnancy may be associated with low birth weight and impaired fetal growth [13,14,15].

AFs are produced mainly by fungi of the genus *Aspergillus*; therefore, fungal colonization of foods favors contamination with these mycotoxins [2]. The establishment and growth of these fungi in foods are influenced by climatic, environmental, and management factors [16]. Mycotoxin contamination affects a substantial proportion of food crops worldwide [17], and environmental and climatic conditions can further influence contamination patterns [16,18]. In addition to their widespread occurrence, AFs are difficult to eliminate from foods because of their high stability, which limits their removal by thermal and chemical treatments [19]. Due to health risks associated with exposure and the presence of these compounds in food, several countries have established regulations defining maximum permitted levels [20]. However, regulatory limits do not eliminate AF-related risk [20], and pregnancy represents a period of increased susceptibility to adverse AF-associated outcomes [21].

Considering that complete decontamination of food is difficult, strategies to reduce mycotoxin bioavailability have been investigated. These include biosorbents, microorganisms or components of their cell walls that can bind AF in the gastrointestinal tract and reduce the fraction available for absorption [22]. Several in vitro studies have demonstrated that lactic acid bacteria (LAB), including *Lacticaseibacillus rhamnosus*, and yeasts such as *Saccharomyces cerevisiae* can bind AF through cell-associated interactions [23,24,25,26]. These microbial approaches form part of broader strategies used to prevent, decontaminate, or minimize mycotoxin toxicity [27]. Heat inactivation of microbial cells does not necessarily abolish AF-binding capacity [24,25,26], while inactivated cell biomass preparations can retain functional properties relevant to food applications [28]. Yogurt is a practical food matrix for incorporating functional microbial cultures [29]. In this context, the combined incorporation of inactivated *L. rhamnosus* and *S. cerevisiae* biomasses into yogurt showed high AFB_1_ adsorption capacity in vitro without compromising the physicochemical or sensory characteristics of the product [30]. More recently, a piglet pilot study using fermented milk with heat-inactivated biomasses of *L. rhamnosus* and *S. cerevisiae* indicated partial protective effects against AF and lower plasma AFP_1_ [31]. However, its effect on AF bioavailability in vivo during the critical period of pregnancy remains uncertain. Accordingly, this work was designed to conduct a first proof-of-concept study aiming at evaluating the effects of gestational exposure to AF on maternal health, fetal development, and the placenta, and the potential mitigating effect of a functional yogurt (FY) containing inactivated *L. rhamnosus* and *S. cerevisiae* biomasses.

## 2. Results

### 2.1. Sample Size, Consumption, and Maternal Weight Gain

At the end of the experimental period, five dams with irregular estrous cycles failed to mate successfully and were excluded. Two rats in the AF group experienced preimplantation pregnancy loss and were included only in analyses of reproductive losses. For all other evaluations, the final number of subjects in each treatment were 8 (Control), 10 (AF) 8 (FY) and 9 (AF + FY).

**Figure 1:**
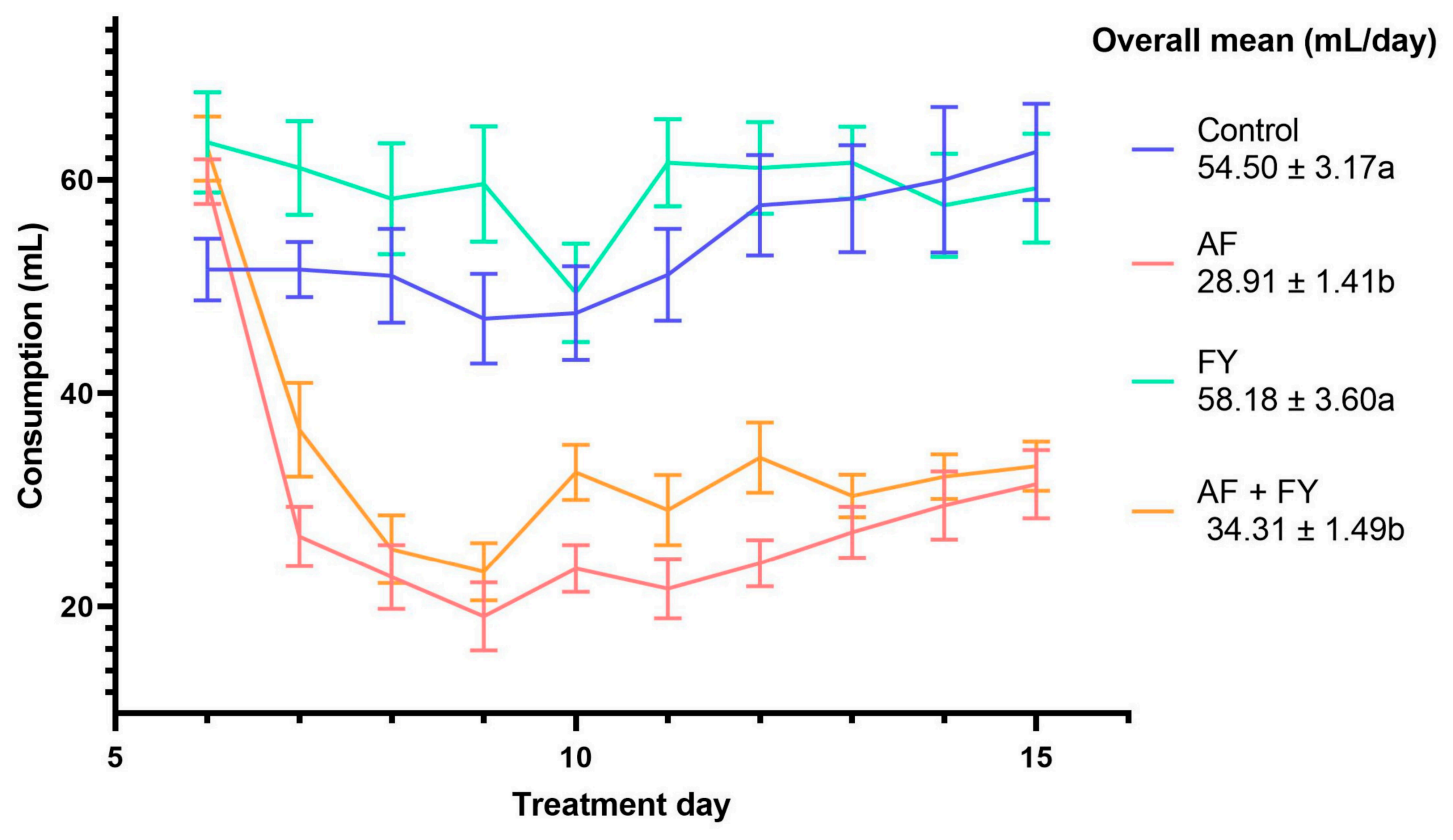
Mean daily intake of the water-yogurt mixture by pregnant Wistar rats during the experimental period from gestational day (GD) 6 to GD15. Points represent daily means and bars represent the standard error of the mean. Control (*n* = 8), aflatoxins (AF; sum of AFB_1_, AFB_2_, AFG_1_ and AFG_2_: 1.0 mg/kg/day) (*n* = 10), functional yogurt (FY) (*n* = 8), and AF + FY (*n* = 9). Overall means followed by different letters differ significantly (*p* < 0.05).

Figure 1 presents the mean daily intake of the water–yogurt mixture by pregnant Wistar rats during the experimental period. The overall mean intake of the water–yogurt mixture was significantly lower in the AF group (28.91 mL/day) than in the Control group (54.50 mL/day). The overall mean intake of animals receiving the FY was 58.18 mL/day, which did not differ from the Control group. Intake in the AF + FY group was 34.31 mL/day, numerically higher than in the AF group but not statistically different from it.

Over the entire gestational period, total maternal weight gain did not differ significantly among groups (*p* > 0.05). However, during the treatment period (GD6–GD15), the AF group gained 11.14 g, significantly less than the Control and FY-receiving groups, as shown in Table 1. The AF + FY group gained 20.11 g and showed an intermediate result, with no significant difference from either the AF group or the Control and FY groups.

### 2.2. Reproductive Outcomes

**Figure 2:**
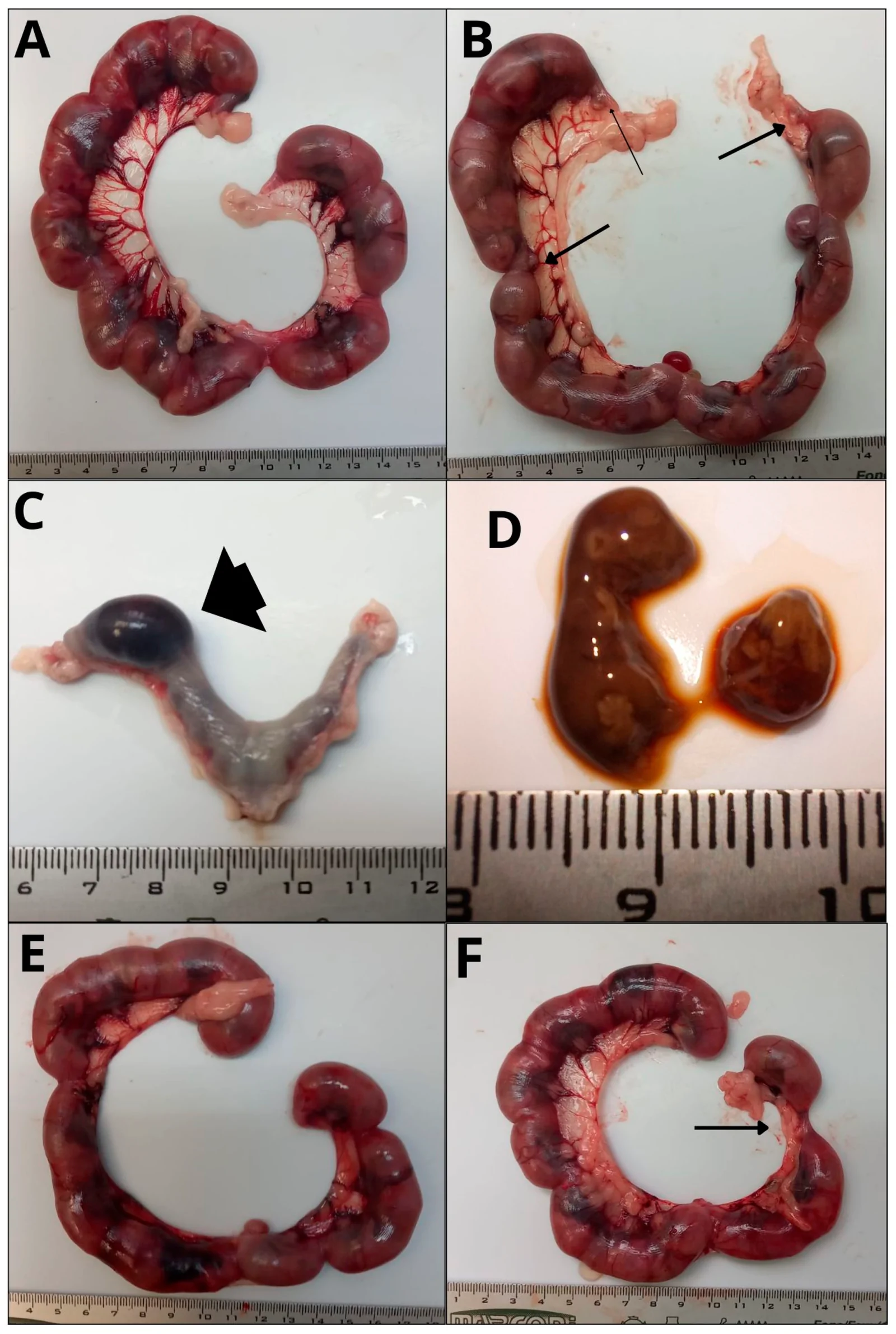
Macroscopic features of gravid uteri and gestational alterations observed in the different experimental groups. (**A**) Control group. (**B**) Aflatoxin (AF; sum of AFB_1_, AFB_2_, AFG_1_ and AFG_2_: 1.0 mg/kg/day) group, with embryofetal resorption sites (arrows). (**C**) Severe late resorption in the AF group (arrowhead). (**D**) Macerated fetus from the uterus shown in C. (**E**) Functional yogurt (FY) group. (**F**) AF + FY group, with an embryofetal resorption site (arrow). Arrows indicate early resorptions; the arrowhead indicates late resorption.

Rats in the AF group were the only showing preimplantation pregnancy loss, with two occurrences among 12 dams evaluated for this outcome. Preimplantation loss was determined by positive vaginal cytology, initial weight gain consistent with pregnancy, and the presence of ovarian corpora lutea associated with the absence of fetuses. Uterine weight, number of live fetuses, and total number of resorptions did not differ significantly among groups, as shown in Table 2. Numerically, the AF group had a lower mean number of live fetuses, lower uterine weight, and a higher number of resorptions. The only late resorption recorded during the experiment occurred in this group. These gestational alterations are shown in Figure 2, including embryofetal resorption sites. In the AF + FY group, these parameters remained close to or intermediate between those of the other groups, with no significant differences.

### 2.3. Fetal and Placental Morphometry

The fetal and placental morphometric parameters evaluated are presented in Table 3. The mean fetal weight in the AF group was 3.21 g, significantly lower than the value (3.91 g) observed in the Control group. The AF + FY group had a mean fetal weight of 3.38 g, an intermediate value that did not differ significantly from either the AF or Control group. The FY group had a mean fetal weight of 3.90 g, similar to the Control group. Body length showed a significant overall difference by ANOVA (*p* = 0.0395), but Tukey’s post hoc test did not identify significant pairwise differences among treatments. In absolute terms, the AF group had the shortest body length (3.39 cm), followed by the AF + FY group (3.42 cm), the Control group (3.62 cm), and the FY group (3.67 cm). Tail length did not differ significantly among groups, although the AF group had the lowest numerical value. Placental weight also did not differ significantly among groups.

### 2.4. Fetal Macroscopic and Histopathological Evaluation

Three fetal deaths were recorded: two in the AF group and one in the AF + FY group. Macroscopically, two of these fetuses had diffusely reddish skin, and one fetus from the AF group had cervical edema. Histopathological analysis revealed no significant alterations in the fetal organs evaluated. Likewise, no histopathological alterations were identified in the placentas examined.

### 2.5. Fetal Skeletal Evaluation

**Figure 3:**
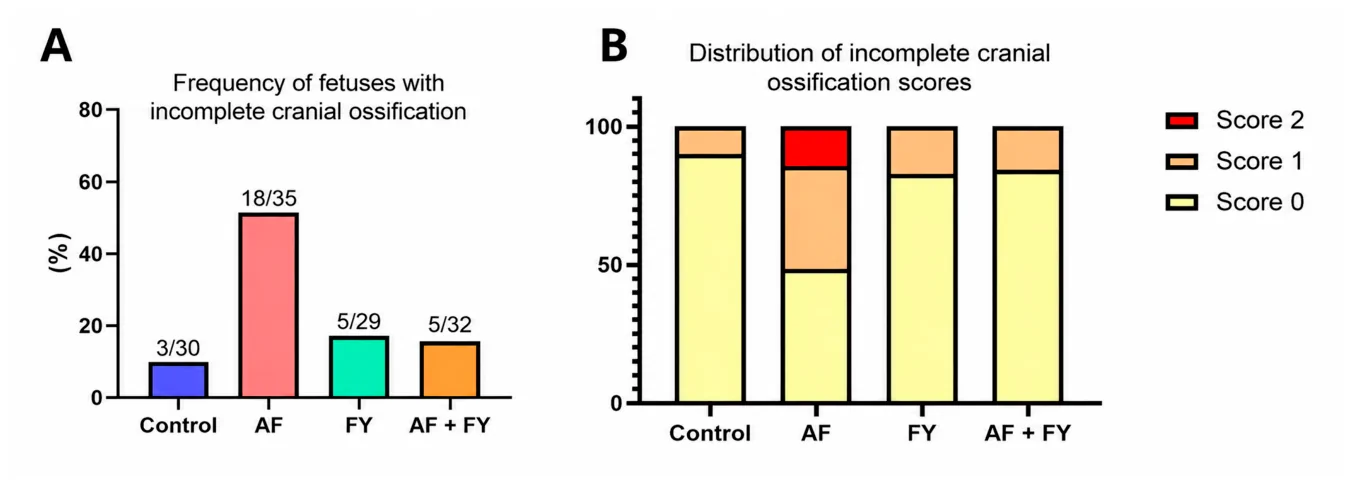
Frequency and severity distribution of delayed cranial ossification in the different experimental groups. (**A**) Frequency of fetuses with incomplete cranial ossification. (**B**) Percentage distribution of ossification scores. AF: aflatoxins (sum of AFB_1_, AFB_2_, AFG_1_ and AFG_2_: 1.0 mg/kg/day); FY: functional yogurt.

**Figure 4:**
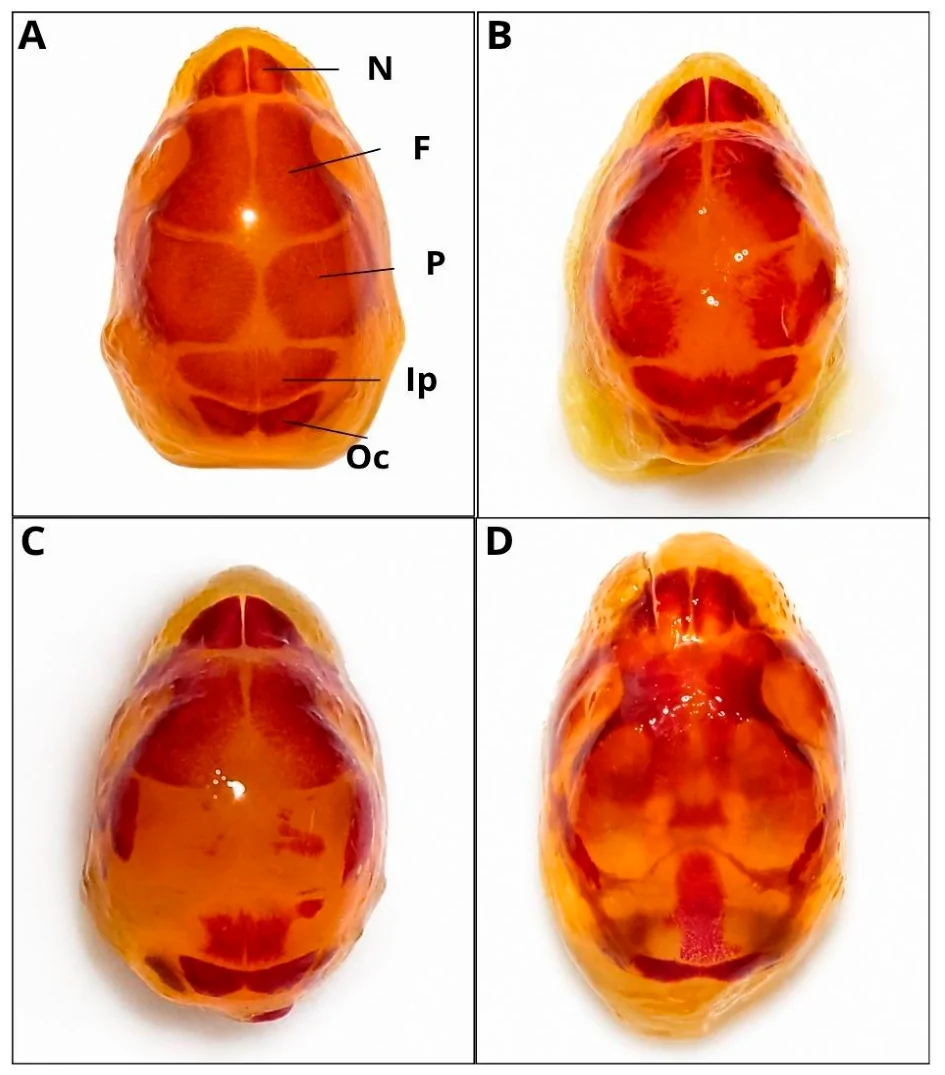
Cranial ossification patterns in cleared and Alizarin Red S-stained fetuses. (**A**) Preserved ossification pattern in the Control group, identifying the nasal (N), frontal (F), parietal (P), interparietal (IP), and occipital (Oc) bones. (**B**) Delayed ossification of the frontal, parietal, and interparietal bones in the aflatoxin (AF; sum of AFB_1_, AFB_2_, AFG_1_ and AFG_2_: 1.0 mg/kg/day) + functional yogurt (FY) group. (**C**) Marked delayed ossification of the parietal bones in the AF group. (**D**) Marked delayed ossification of the frontal, parietal, and interparietal bones in the AF group, with absence of ossification in the interparietal region (score 2).

Skeletal evaluation revealed delayed ossification of the cranial bones, with a marked difference among groups, as shown in Figure 3. Delayed ossification was observed in 3/30 (10.0%) fetuses in the Control group, 18/35 (51.4%) in the AF group, 5/29 (17.2%) in the FY group, and 5/32 (15.6%) in the AF + FY group. Because individual affected fetuses could not be retrospectively linked to their respective litters, these fetal-level frequencies are presented descriptively and were not subjected to inferential comparisons treating individual fetuses as independent observations. Sensitivity analysis based on the known number of fetuses examined from each dam showed that the 18 affected fetuses in the AF group necessarily originated from at least five of the nine litters contributing fetuses to the skeletal evaluation. Considering all feasible within-litter allocations, the mean litter-level proportion of affected fetuses ranged from 50 to 52.8% in the AF group and from 13.9 to 18.5% in the AF + FY group. Thus, the pronounced difference between these groups could not be explained solely by concentration of the abnormalities within one or a few litters. The most severe cases, classified as score 2 and characterized by loss of bone contours, occurred exclusively in the AF group, affecting 5 of the 35 fetuses evaluated. Among the 31 fetuses with some degree of delayed ossification, 96.8% of cases had parietal bones affected, followed by the interparietal (58.1%), frontal (29.0%), and occipital bones (12.9%), as presented in Figure 4.

### 2.6. Placental Immunostaining for Placental Growth Factor (PlGF)

**Figure 5:**
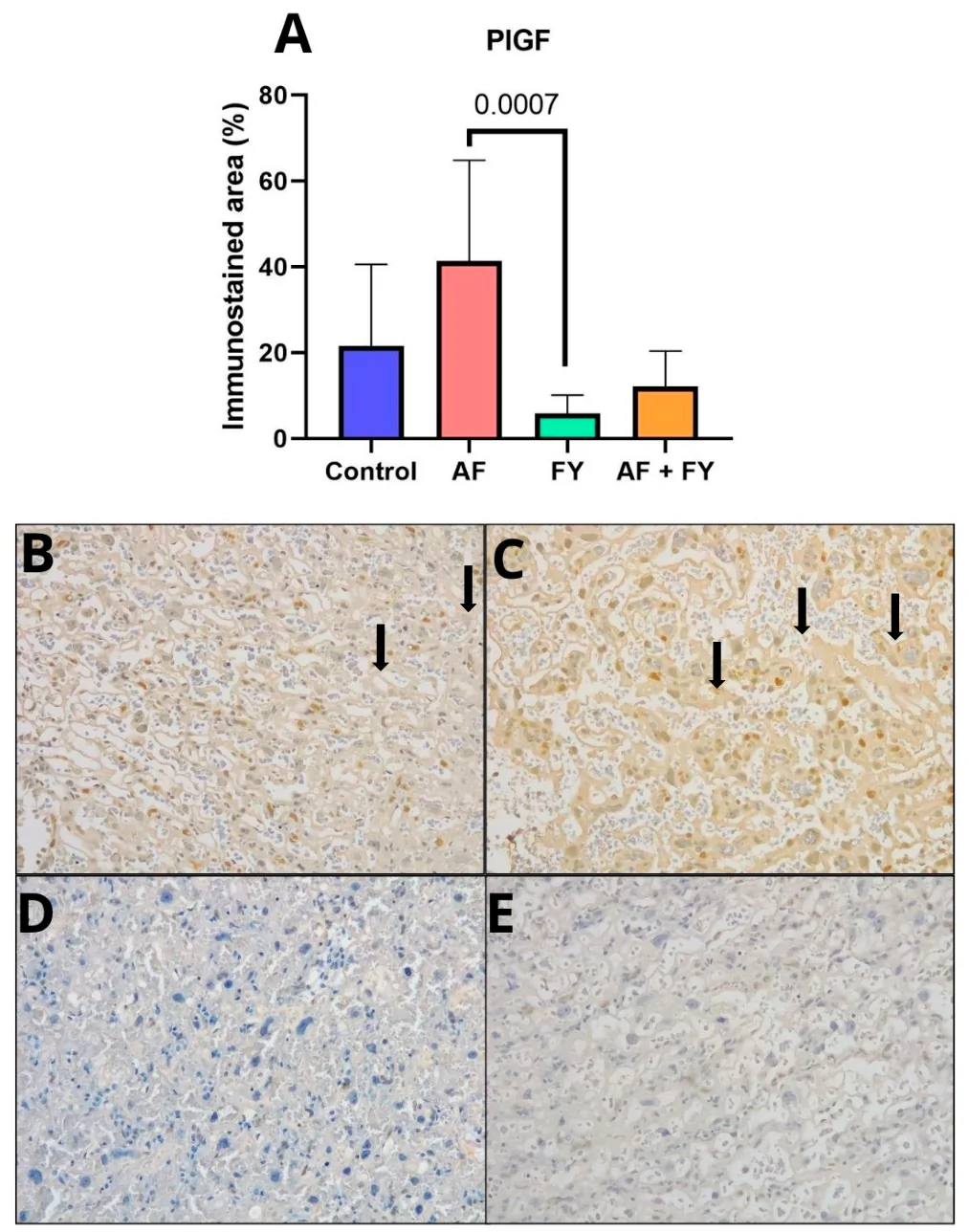
Placental PlGF immunostaining in the different experimental groups. (**A**) Quantification of the DAB-immunopositive area. (**B**–**E**) Representative photomicrographs of the placental labyrinth zone from the Control, aflatoxins (AF; sum of AFB_1_, AFB_2_, AFG_1_ and AFG_2_: 1.0 mg/kg/day), functional yogurt (FY) and AF + FY groups, respectively. Control (*n* = 8), AF (*n* = 9), FY (*n* = 7), and AF + FY (*n* = 9). Magnification: 200×. Black arrows indicate positive immunostaining.

PlGF immunostaining differed significantly among groups (Kruskal–Wallis, *p* = 0.001). The AF group had the highest mean percentage of immunopositive area (41.36 ± 23.46%), whereas the Control, FY, and AF + FY groups had values of 21.66 ± 18.93, 5.82 ± 4.28, and 12.10 ± 8.28%, respectively (Figure 5). Dunn’s post hoc test showed greater immunostaining in the AF group than in the FY group (*p* = 0.0007). Although the AF + FY group had a numerically lower value than the AF group, this comparison did not reach statistical significance after adjustment for multiple comparisons.

### 2.7. Determination of Aflatoxins and Their Metabolites

AFs and their metabolites remained below the limit of quantification in serum, maternal liver, placenta, and fetal liver samples from the Control and FY groups. Chromatographic analyses therefore focused on the AF and AF + FY groups.

**Figure 6:**
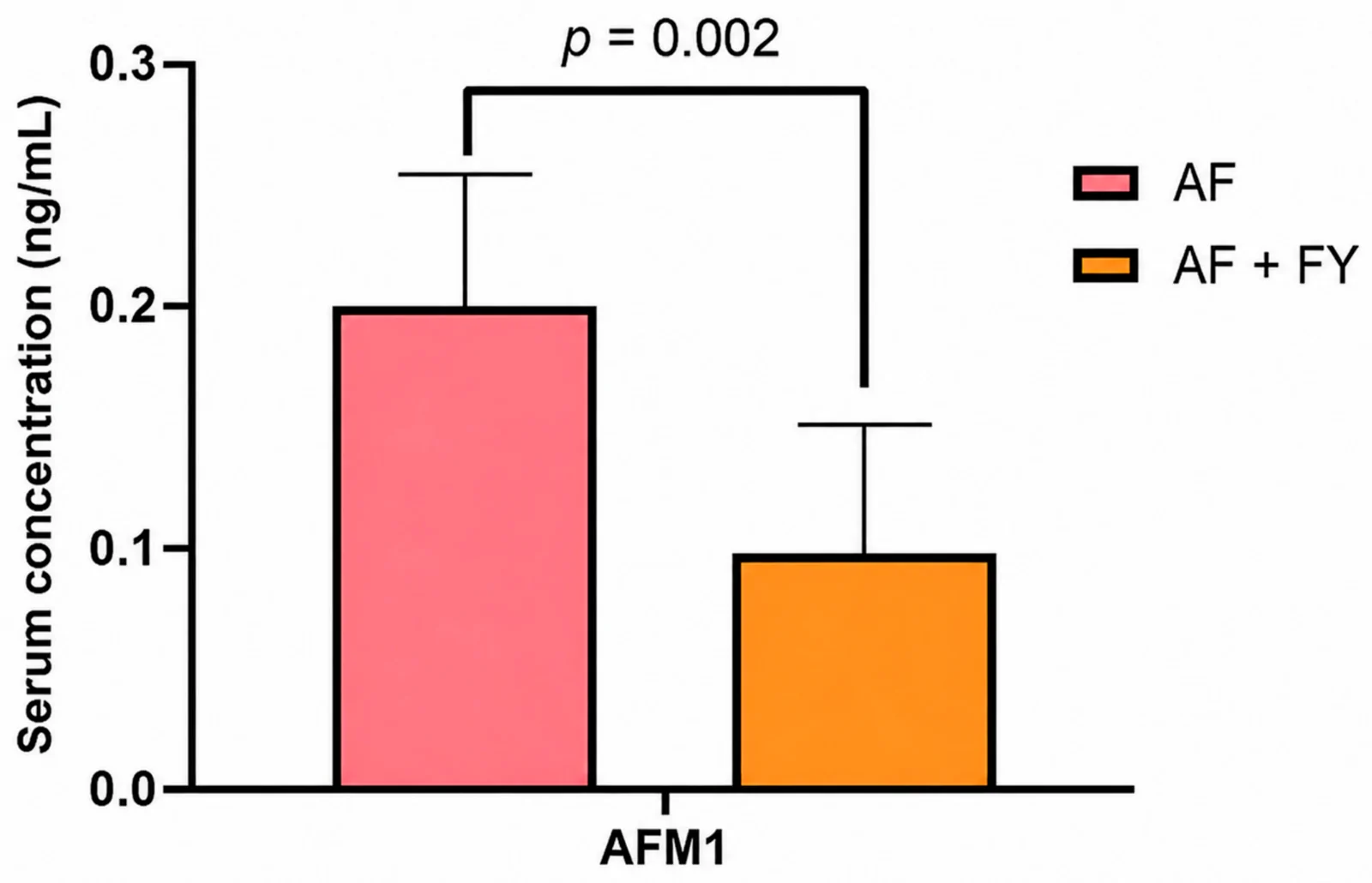
Serum concentrations of aflatoxin M_1_ (AFM_1_) in Wistar rats receiving aflatoxins (AF; sum of AFB_1_, AFB_2_, AFG_1_ and AFG_2_: 1.0 mg/kg/day) and AF + functional yogurt (FY). Bars represent the mean ± standard deviation (AF, *n* = 10; AF + FY, *n* = 8). AFM_1_ concentrations were compared using the Mann–Whitney test, and a significant difference was observed between the groups (*p* = 0.002).

In maternal serum, AFQ_1_ was not identified at quantifiable levels. However, AFP_1_ was detected only in the AF group, in three of the ten animals evaluated, at concentrations ranging from 0.11 to 0.12 ng/mL. AFM_1_ concentrations differed significantly between groups (*p* = 0.002), with a mean of 0.199 ± 0.0549 ng/mL in the AF group and 0.0967 ± 0.0540 ng/mL in the AF + FY group (Figure 6). In addition, AFM_1_ was quantified in all samples from the AF group but in only five of the eight samples from the AF + FY group.

AFB_1_ and its metabolites were quantified more frequently in maternal liver samples from the AF group, as presented in Table 4. Non-metabolized AFB_1_ and AFP_1_ were the most frequently identified compounds, each present in six of the ten samples evaluated (60%), with mean concentrations of 0.84 and 0.26 ng/g, respectively. AFM_1_ was quantified in three samples (30%), whereas AFB_2_ and AFG_1_ were found in two samples each (20%), and AFG_2_ in one sample (10%). AFQ_1_ remained below the limit of quantification (LOQ: 0.09 ng/g) in all samples. In the AF + FY group, only one of the eight samples had quantifiable concentrations of AFM_1_, AFB_1_, and AFG_1_, while all other analytes remained below the LOQ in all samples evaluated. Considering all analytes evaluated, at least one type of AF was quantified in 8 of 10 samples from the AF group, compared with 1 of 8 samples from the AF + FY group. Fisher’s exact test showed a statistically significant difference in the frequency of quantification between groups (*p* = 0.015).

Because of the limited amount of tissue available, placentas from the same dam were pooled. In placental samples from rats in the AF group, AFP_1_ was the most frequently identified analyte, being quantified in four of the five samples evaluated (80%) at a mean concentration of 0.12 ng/g and a range of 0.11–0.13 ng/g (Table 5). AFB_1_ was quantified in two samples (40%), with a mean concentration of 0.19 ng/g and a range of 0.12–0.25 ng/g. All other analytes remained below the limit of quantification. In the AF + FY group, none of the analytes evaluated were present at concentrations above the LOQ (0.08, 0.09, 0.13, 0.09, 0.18, 0.10 and 0.19 ng/g for aflatoxins M_1_, P_1_, Q_1_, B_1_, B_2_, G_1_ and G_2_, respectively).

As with the placentas, fetal livers from the same litter were pooled. In the AF group, AFP_1_ was the most frequently quantified analyte in fetal liver and was present in four of the nine pools evaluated (44%), with a mean concentration of 0.12 ng/g and a range of 0.09–0.18 ng/g (Table 5). AFM_1_ and AFB_1_ were each quantified in one pool (11% of samples), at concentrations of 0.12 and 0.81 ng/g, respectively. All other analytes remained below the limit of quantification, and no analyte was quantified in the AF + FY group.

## 3. Discussion

AF exposure affected maternal performance during the treatment period. Intake of the yogurt–water mixture and maternal weight gain were significantly lower in the AF group than in the Control and FY groups. Animals receiving AF + FY showed intermediate values for both parameters, although these differences were not significant. These intermediate values may therefore be related to consumption of the FY. Reduced maternal weight gain has previously been reported in pregnant rats exposed to AFB_1_ [10,12]. Reduced body-weight gain and feed consumption have also been observed in AF-exposed piglets [31], while AFB_1_-related adverse effects on productive performance have been reported in broilers [32]. This response is likely multifactorial. AFB_1_ can reduce food intake [33] and disrupt hypothalamic neuropeptide pathways involved in feeding regulation [34]. Therefore, the lower consumption observed during AF exposure may have contributed to the reduced maternal weight gain. At the same time, a direct contribution of systemic AF toxicity cannot be excluded. The intermediate response in the AF + FY group suggests partial attenuation, although the present data do not establish a significant protective effect on maternal weight gain.

A similar pattern was observed for reproductive outcomes. No significant differences were observed among groups; however, the most severe events were recorded in AF-exposed animals. All three fetal deaths occurred in these groups, including two in the AF group and one in the AF + FY group. The two preimplantation pregnancy losses were restricted to animals exposed to AF alone. Likewise, the only late resorption occurred in this group and was accompanied by resorption of the entire litter. These observations are consistent with previous evidence of AFB_1_-associated reproductive and developmental toxicity [10,12,35]. In particular, Ibeh and Saxena [35] reported increased fetal resorption, implantation loss, and intrauterine death after AFB_1_ exposure. Although our findings were not statistically significant, the absence of these more severe outcomes in the AF + FY group, except for one fetal death, suggests that the FY may have contributed to attenuating reproductive toxicity. However, the data do not support a preventive effect.

Consistent with the literature, fetal weight was significantly lower in the AF group than in the Control group [9,10,11,36]. However, the AF + FY group did not differ significantly from either the Control or AF group, yielding an intermediate value that may indicate attenuation of AF effects on the offspring of animals supplemented with the FY. A similar pattern was observed for the other morphometric parameters, for which the AF + FY group generally showed values intermediate between the AF group and the Control and FY groups, although these differences did not reach statistical significance. The skeletal findings showed a pronounced descriptive difference between the AF and AF + FY groups, with delayed cranial ossification observed in 51.4 and 15.6% of the examined fetuses, respectively. Sensitivity analysis based on the known number of fetuses examined per dam indicated that the higher frequency in the AF group could not be explained by abnormalities confined to only one or a few litters. Nevertheless, the absence of individual fetus-to-litter correspondence prevented formal litter-level inferential analysis and should be considered a limitation of the skeletal evaluation. Delayed ossification is among the most frequently reported skeletal alterations following prenatal AF exposure [9,10,11,12]. The marked reduction in its frequency and severity with FY supplementation therefore indicates a substantial attenuation of the skeletal response. This interpretation is further supported by the lower internal AF burden observed in the same group, linking reduced exposure with a less pronounced developmental effect. However, delayed ossification should not be interpreted as a permanent skeletal malformation. It is generally considered a developmental variation because minor delays in ossification may be repaired through postnatal skeletal remodeling [37]. Variability in ossification status is also documented in Wistar rat fetuses near term [38], which is consistent with the occurrence of some delayed ossification in the Control and FY groups. Its toxicological relevance in the present study arises from the marked increase after AF exposure and the occurrence of severe lesions only in this group. Several mechanisms may contribute to impaired skeletal development. AFB_1_ has been reported to alter calcium utilization [39] and to down-regulate vitamin D receptor expression in vitro [40], both of which may impair pathways relevant to skeletal mineralization. Additional experimental evidence indicates that AFB_1_ may impair bone mineralization through disturbances in vitamin D and calcium/phosphorus homeostasis [41]. Maternal toxicity during organogenesis may also have indirectly affected fetal growth and mineralization. These mechanisms were not evaluated here and should therefore be considered biologically credible explanations rather than demonstrated pathways.

Placental morphology appeared less affected under the conditions of this experiment. Placental weight and histological architecture did not differ among groups. The placenta plays a pivotal role in fetal growth and is a relevant target in developmental toxicology [42]. In contrast, PlGF immunostaining presented a distinct response, with the greatest immunopositive area in the AF group and the lowest in the FY group. PlGF is an angiogenic factor involved in placental vascular development, and altered placental PlGF expression has been reported in intrauterine growth restriction [43]. In our study, however, placental PlGF staining was greatest in the AF group, which was also the group with the smallest fetuses, whereas the FY group, whose fetal weights were close to those of the Control group, showed the lowest staining. One possible explanation for this finding is a compensatory angiogenic response in the placentas of rats exposed to AF after the exposure period. This hypothesis may be consistent with the observation that, after AF administration ended, females in this group resumed gaining weight and approached the other groups. However, the data obtained in this study do not allow this relationship to be established. Interestingly, the FY group showed the lowest PlGF immunostaining, followed by the AF + FY group. This pattern raises the possibility that FY consumption may also have influenced the placental response involving PlGF, although additional studies are needed to clarify this finding.

The analytical results on AF biomarkers determined in serum and tissues provide more direct evidence that FY reduced internal AF exposure. Maternal serum AFM_1_ concentrations were significantly lower in the AF + FY group than in animals exposed to AF alone. AFB_1_ and their metabolites were also quantified less frequently in maternal liver following FY supplementation. In addition, none of the investigated compounds were present above their LOQ in placental or fetal-liver samples from the AF + FY group. In contrast, AFP_1_ and AFB_1_ were quantified in placenta, while AFP_1_, AFM_1_, and AFB_1_ were detected in fetal liver from animals receiving AF alone. These results demonstrate that maternal exposure resulted in the presence of AF-related compounds at the maternal–fetal interface and in fetal tissue, whereas FY supplementation markedly reduced their measurable occurrence. This evidence of fetal exposure is consistent with recent human biomonitoring in which AFM_1_ was quantified in serum and liver samples from stillborns and neonates, supporting prenatal transfer of AF-related compounds [44].

AFM_1_, AFP_1_, and AFQ_1_ are established phase I products of AFB_1_ biotransformation, although their relative formation is species-dependent [5,45]. The occurrence of AFM_1_ and AFP_1_ in the biological matrices evaluated here is therefore consistent with the expected metabolism of AFB_1_ [5,45]. Importantly, values below the LOQ should not be interpreted as complete absence of exposure. They indicate that concentrations were below the quantitative capability of the analytical method at the time of sampling. Nevertheless, the significant reduction in circulating AFM_1_, together with the lower frequency of quantifiable compounds in maternal liver and the absence of measurable residues in placenta and fetal liver, provides a consistent toxicokinetic pattern of reduced systemic exposure following FY supplementation. This reduction is biologically compatible with gastrointestinal adsorption of AF by the microbial biomasses incorporated into the yogurt. Inactivated *L. rhamnosus* and *S. cerevisiae* have previously shown high in vitro AFB_1_-binding capacity through interactions with cell-wall components [30]. Such binding could decrease the fraction of toxin available for intestinal absorption and consequently reduce its systemic distribution [22,30]. Consistent with this interpretation, Freire et al. [31] reported partial organ-specific protection and lower plasma AFP_1_ in piglets receiving fermented milk with the same heat-inactivated biomasses, although AFM_1_ was not consistently reduced. Reductions in internal AF biomarkers or tissue residues have also been reported with other adsorbent strategies in pigs [46,47]. However, gastrointestinal binding itself was not directly measured in the present study. Therefore, our results support an in vivo biosorption effect rather than conclusively demonstrating the underlying process.

Taken together, the developmental and toxicokinetic results show a coherent response. FY supplementation was associated with lower systemic AF exposure and a pronounced reduction in delayed cranial ossification. These two outcomes provide the strongest evidence of mitigation in the present study. In contrast, maternal weight gain, reproductive parameters, and most fetal morphometric outcomes showed mainly intermediate numerical responses without significant differences from animals exposed to AF alone. Thus, the protective effect of FY appears to be endpoint-dependent rather than complete. Overall, the findings support the potential of FY containing inactivated *L. rhamnosus* and *S. cerevisiae* as a strategy to reduce AF bioavailability and attenuate selected manifestations of maternal–fetal toxicity.

## 4. Conclusions

Under the conditions of this experiment, supplementation with a FY containing *L. rhamnosus* and *S. cerevisiae* in pregnant rats exposed to AF resulted in lower circulating AFM_1_ levels than in the non-supplemented group. In parallel, 15.6% of fetuses in the group exposed to AF and supplemented with FY showed cranial ossification defects, compared with 51.4% in the group exposed only to AF, indicating a possible protective effect of the FY against AF-induced skeletal damage. Regarding gestational and fetal outcomes, although FY supplementation numerically attenuated the adverse effects, these results did not differ statistically from those observed in the non-supplemented group. The study also showed that FY administration did not adversely affect maternal weight gain, fetal morphometry, or skeletal development compared with the Control group. Placental PlGF immunostaining showed a greater immunopositive area in the AF group, with a significant difference relative to the FY group. Thus, AF exposure appeared to be associated with increased PlGF staining in the placenta, which may reflect a compensatory response involving this protein. The opposite pattern was observed in the group that received FY alone. These findings warrant further investigation to better understand the effects of AF and FY on placental PlGF. Finally, considering the parameters evaluated, FY consumption appeared to have beneficial effects in animals exposed to AF, supporting its potential to act as an in vivo biosorbent to attenuate the adverse effects of these toxins during pregnancy. As this was an initial proof-of-concept study, further studies are needed to determine whether this effect is maintained at different inclusion levels. Future studies should also directly investigate aflatoxin biosorption and fecal elimination, assess the individual contribution of *L. rhamnosus* and *S. cerevisiae*, and examine whether the skeletal alterations observed during fetal development persist after birth. Additional studies combining skeletal evaluation with molecular markers of bone development and placental vascular responses may also help clarify the mechanisms involved.

## 5. Materials and Methods

### 5.1. Ethical Aspects, Animals, and Housing Conditions

All experimental procedures were approved by the Animal Experimentation Ethics Committee of the Ribeirão Preto Medical School, University of São Paulo (CEUA/FMRP-USP; protocol 1053/2022) and followed national and international standards for animal welfare and biosafety.

Forty-two Wistar rats obtained from the Central Animal Facility of the University of São Paulo were used. The animals were maintained at 22 ± 1 °C in isolators with an air-exchange system under a 12 h light/dark cycle, with food and water available ad libitum. The females were acclimated until 10 weeks of age, when they were used for breeding. Copulation was confirmed by vaginal cytology, and the presence of spermatozoa was designated as GD0. Pregnant females were housed individually.

### 5.2. Preparation of Aflatoxins and Functional Yogurt

The AF mixture was produced from *Aspergillus parasiticus* NRRL 2999 cultured in rice, as described elsewhere [31]. Briefly, the *A. parasiticus* strain was inoculated onto potato dextrose agar (PDA) plates (Interlab, São Paulo, Brazil) and incubated at 25 °C for 5 days. After this period, 76 g of sterile rice was weighed in an Erlenmeyer flask and inoculated with small pieces of the seeded PDA. The inoculated flasks were placed in a horizontal shaker (Tecnal, Piracicaba, Brazil), which was kept at 250 rpm and incubated for 1 week in the dark at room temperature. The flasks with AF-culture material were placed in a chemical hood, and 35–40 mL of chloroform was carefully added in each flask to stop the growth of the fungi. The mixture was transferred onto stainless steel trays and kept in a forced air oven at 40 °C for 48 h until dryness. After drying, the AF-culture material was milled and extracted with methanol:water (80:20, *v*/*v*), which was subsequently evaporated under N_2_ flow at 55 °C until dryness. The residue was resuspended in dimethyl sulfoxide (DMSO) and submitted to AF quantification according to Freire et al. [31]. The solution had a total AF concentration of 1.372 mg/mL and consisted of AFB_1_ (68.2%), AFG_1_ (28.5%), AFB_2_ (2.8%), and AFG_2_ (0.5%). The dose administered to the exposed groups was 1.0 mg/kg/day of total AF.

The microbial biomasses used in this study were lyophilized commercial strains of *L. rhamnosus* (HOWARU^®^ Rhamnosus LYO 40 DCU, Danisco Ltd., São Paulo, Brazil) and *S. cerevisiae* (Fermentis K-97, SafAle, Bruggeman, Gent, Belgium), both containing 1.0 × 10^10^ viable cells/g before inactivation. These biomasses were autoclaved at 121 °C for 10 min before added to the FY. Yogurt preparation followed the protocol as described by Pires et al. [30]. Briefly, 5 L of ultra-high temperature skim milk were purchased from local supermarkets, mixed in a 15-L yogurt-producing vat and standardized by adding skim milk powder to reach 12.0% total solids (*w*/*v*) content. The heat-inactivated biomasses were added to the standardized milk at 4.0% *L. rhamnosus* (*w*/*v*) and 4.0% *S. cerevisiae*, (*w*/*v*), followed by mixing for 10 min. The mixture was heated-treated at 90 °C for 15 min, then reduced to 42 °C for the inclusion of the starter culture containing *Streptococcus thermophilus* and *Lactobacillus delbrueckii* ssp. *bulgaricus* (Yo-Flex, Chr. Hansen, Horsholm, Denmark) at 2.0% inoculum. After incubation at 42 °C until reaching a pH value of 4.5, the FY was cooled to 10 °C, transferred to 1 L polyethylene bottles and stored at 5 °C for a maximum of 15 days until use. Control yogurt samples were also prepared without any microbial biomass, following the same procedures described above. For administration to the animals, the FY was mixed with water at a ratio of 25% yogurt to 75% water. Thus, in the groups receiving FY, the final mixture contained 1% biomasses of both *L. rhamnosus* and *S. cerevisiae*.

### 5.3. Experimental Design

**Figure 7:**
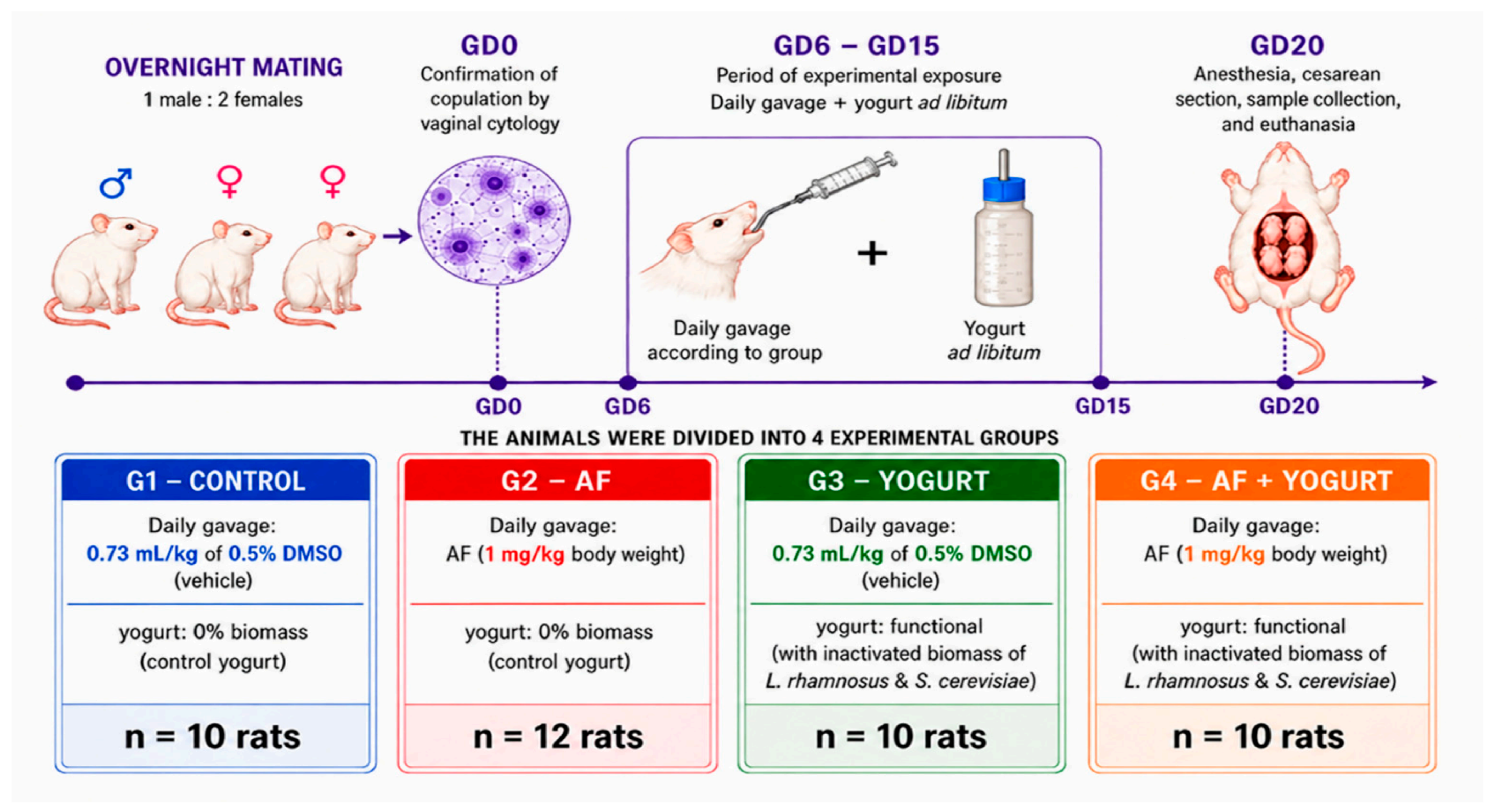
Experimental design. Graphical illustration showing that the rats were allocated into four groups and treated daily from gestational day (GD) 6 to GD15 with dimethyl sulfoxide (DMSO) or aflatoxins (AF; sum of AFB_1_, AFB_2_, AFG_1_ and AFG_2_: 1.0 mg/kg/day), in combination with control or functional yogurt (FY). On GD20, cesarean sections, sample collection, and euthanasia were performed.

From gestational day 6 to 15 (GD6–GD15), corresponding to the period of organogenesis, the rats were randomly allocated to four experimental groups: Control, which received 0.5% (*v*/*v*) DMSO by gavage and water containing control yogurt; AF, which received 1.0 mg/kg/day of total AF by gavage and water containing control yogurt; FY, which received 0.5% (*v*/*v*) DMSO and water containing FY with inactivated biomass; and AF + FY, which received total AF (1.0 mg/kg/day) and water containing FY. The water–yogurt mixtures were provided ad libitum, prepared daily, and replaced the following morning. On GD20, the rats underwent cesarean section and were subsequently euthanized under deep anesthesia (Figure 7).

### 5.4. Consumption of the Water–Yogurt Mixture and Maternal Weight Gain

The amount of water–yogurt mixture consumed by each dam was measured daily throughout the treatment period, from GD6 to GD15. Weight gain was evaluated over the GD0–GD6, GD6–GD15, and GD15–GD20 intervals, as well as over the entire period from GD0 to GD20.

### 5.5. Cesarean Section, Reproductive Outcomes, and Fetal and Placental Morphometry

On GD20, pregnant rats were anesthetized intraperitoneally with ketamine (100 mg/kg) and xylazine (10 mg/kg) and subjected to a midline abdominal incision to expose the uterine horns. The uteri were examined and weighed. The numbers of live fetuses, embryofetal resorptions, and preimplantation pregnancy losses were recorded.

Fetuses were examined macroscopically and subjected to morphometric evaluation, including body weight, body length from head to tail, and tail length. Placentas were also weighed.

### 5.6. Fetal and Placental Histopathology

Fetuses designated for histopathological examination were dissected for collection of the heart, lungs, liver, kidneys, gonads, and spleen. Fetal and placental samples were fixed in buffered formalin for 48 h, routinely processed for paraffin embedding, sectioned at 5 µm, and stained with hematoxylin and eosin. Slides were evaluated for histopathological alterations.

### 5.7. Fetal Skeletal Evaluation

Fetuses with preserved structural integrity were cleared in 1% KOH for 72 h and subsequently stained with 0.005% alizarin red S (Sigma-Aldrich, Saint Louis, MO, USA) for 48 h. After successive steps in KOH and glycerol, the specimens were stored in pure glycerol. The entire fetal skeleton was initially examined using a magnifying lens and ImageJ software for absent or deformed bones and delayed ossification. Since ossification abnormalities were identified exclusively in the cranial bones, the detailed evaluation focused on the frontal, parietal, interparietal, and occipital bones. Ossification defects were classified as score 0, no defects; score 1, defects present with bone contours still identifiable; and score 2, loss of architecture with inability to adequately identify the bone contour.

### 5.8. Placental Immunohistochemistry for PlGF

For PlGF evaluation, one placenta sample was randomly selected from each dam. Placental sections mounted on silanized slides were deparaffinized, rehydrated, and subjected to antigen retrieval in citrate buffer, pH 6.0, under steam for 40 min. After blocking endogenous peroxidase activity and nonspecific proteins, the slides were incubated with an anti-PlGF antibody (Affinity Biosciences, Cincinnati, OH, USA; AB-DF6579; 1:100), followed by the Novolink Polymer detection system and development with 3,3′-diaminobenzidine (DAB). For quantification, five randomized fields from each sample were obtained at 20× magnification. The fields were analyzed using ImageJ version 1.54p (National Institutes of Health, Bethesda, MD, USA) by color deconvolution with the DAB H vector, and results were expressed as the percentage of DAB-immunopositive area.

### 5.9. Determination of Aflatoxins and Their Metabolites

Analytical-grade reagents and Milli-Q water (Millipore, Bedford, MA, USA) were used, together with HPLC-grade acetonitrile and methanol (JT Baker, Xalostoc, Mexico). AF standards (AFB_1_, AFB_2_, AFG_1_, AFG_2_, AFM_1_, AFP_1_, and AFQ_1_) were purchased from Sigma (Sigma, St. Louis, MO, USA). An isotopically labeled internal standard (IS) of [^13^C_17_]-AFB_1_ (Sigma, St. Louis, MO, USA) was also used, with its individual stock solution prepared in acetonitrile:water (1:1, *v*/*v*) at 5 ng/mL.

Serum, placenta, and hepatic tissue samples were extracted following the procedures as described by Muñoz-Solano and González-Peñas [48], with some modifications. Briefly, 450 µL of serum and 0.5 ± 0.005 g of placenta or homogenized hepatic tissue were mixed with 5 µL of the AFB_1_ IS solution and 50 µL of β-glucuronidase/arylsulfatase type H-3 from *Helix pomatia* (Sigma-Aldrich, Cotia, Brazil) in a 2 mL microcentrifuge tube. The mixture was incubated overnight at 37 °C and then transferred to a 3 mL syringe coupled to a Captiva EMR-Lipid column (Agilent Technologies, Barueri, Brazil). Next, 1.5 mL of acetonitrile containing 1% formic acid was added. After 5 min, the solution was passed through the column. The tube was washed with 500 µL of acetonitrile/water (75:25, *v*/*v*) containing 1% formic acid, and the wash solution was also passed through the column. The eluate was evaporated to dryness at 60 °C under N_2_ flow at 0.13 bar, using a MultiVap-54 system (LabTech, Sorisole, Italy). The residue was reconstituted in 450 µL of acetonitrile/water (10:90, *v*/*v*), vortexed, filtered through 0.22 µm PTFE syringe filters (Perfecta, São Paulo, Brazil), and stored at −20 °C until analysis.

Extracts were analyzed liquid chromatography–tandem mass spectrometry (LC-MS/MS), using a Waters Acquity I-Class LC system (Waters, Milford, MA, USA) equipped with a BEH C18 column (2.1 × 50 mm; 1.7 µm) and coupled to a Xevo TQ-S triple-quadrupole MS/MS (Waters, Milford, CT, USA). Five microliters of each sample extract were injected into the LC-MS/MS system. Mobile phase, gradient elution and other chromatographic parameters were set exactly as described by Fermiano et al. [44] and displayed in Supplementary Table S1. Matrix-matched calibration curves were prepared using samples extracts from Controls, previously analyzed and containing non-detectable levels of any AF. Five-point calibration curves were constructed with a mixture of AF standards (AFB_1_, AFB_2_, AFG_1_, AFG_2_, AFM_1_, AFQ_1_ and AFFP_2_) dissolved in water:acetonitrile (9:1, *v*/*v*), at concentrations of 0.625, 1.25, 2.50, 5.00 and 10.0 ng/g. Prior to injection, 20 µL of the IS standard solution was added to 80 µL of the standard solution mixture in each calibration point. AF were identified and quantified using the positive mode in multiple reaction monitoring (MRM) mode. The MRM transitions required for quantification and confirmation, and the optimized MS parameters for each AF analyzed, are presented in the Supplementary Table S2. The limits of detection (LOD) and quantification (LOQ) were calculated based on signal-to-noise ratios of 3:1 and 10:1, respectively, and the respective values are displayed in Supplementary Table S2. Data collection and processing were performed using MassLynx software (v. 4.1).

### 5.10. Statistical Analysis

Dams/litters were considered the experimental unit for maternal, reproductive, fetal morphometric, and placental morphometric outcomes. For fetal and placental morphometry, measurements obtained within each litter were averaged before statistical analysis. Skeletal abnormalities were recorded at the individual fetus level. Because affected fetuses could not be retrospectively linked to their respective dams, a deterministic sensitivity analysis was performed using the known number of fetuses examined per dam and all feasible within-litter allocations of affected fetuses. No inferential *p*-values were derived from this analysis. Data are presented as mean ± standard deviation or standard error of the mean, as appropriate. Comparisons were performed by ANOVA followed by Tukey’s test or by the nonparametric Kruskal–Wallis test followed by Dunn’s test. Data collected over time were evaluated by two-way repeated-measures ANOVA. Frequencies were compared using association tests, including Fisher’s exact test when applicable. Serum AFM_1_ concentrations were compared between the AF and AF + FY groups using the Mann–Whitney test. Values below the LOQ were considered left-censored and replaced with half the LOQ for statistical analysis. Values of *p* < 0.05 were considered statistically significant. Analyses were conducted using GraphPad Prism software (v. 6.0).

## Figures and Tables

**Table 1:** Maternal weight gain in pregnant Wistar rats during different gestational periods and total weight gain ^1^.

Gestational Period	Control	AF	FY	AF + FY	*p*-Value
GD0–GD6	14.25 ± 7.62 ^ab^	7.91 ± 7.16 ^b^	17.10 ± 5.05 ^a^	13.13 ± 6.06 ^ab^	0.0401
GD6–GD15	26.69 ± 10.41 ^a^	11.14 ± 7.88 ^b^	28.33 ± 12.54 ^a^	20.11 ± 11.01 ^ab^	0.0056
GD15–GD20	50.44 ± 16.37	63.57 ± 10.67	59.50 ± 18.14	65.56 ± 11.97	0.1584
GD0–GD20	91.38 ± 19.22	82.62 ± 20.99	104.93 ± 18.68	98.74 ± 16.61	0.0953

^1^ Data are presented as mean ± standard deviation. Control (*n* = 8), aflatoxins (AF; sum of AFB_1_, AFB_2_, AFG_1_ and AFG_2_: 1.0 mg/kg/day) (*n* = 10), functional yogurt (FY) (*n* = 8), and AF + FY (*n* = 9). Data were analyzed by one-way ANOVA followed by Tukey’s post hoc test. Different letters within the same row indicate statistically significant differences among groups (*p* < 0.05). GD: gestational day.

**Table 2:** Reproductive parameters and outcomes in pregnant Wistar rats ^1^.

Parameter	Control	AF	FY	AF + FY	*p*-Value
Uterine weight (g)	52.63 ± 5.27	47.90 ± 17.45	54.16 ± 15.40	57.53 ± 8.10	0.467
Live fetuses	9.83 ± 1.72	9.10 ± 3.54	9.38 ± 3.25	10.78 ± 1.09	0.599
Total resorptions	0.50 ± 0.84	1.70 ± 2.83	0.38 ± 1.06	0.44 ± 0.73	0.419
Dams without implantation, *n*/*N* (%)	0/8 (0)	2/12 (16.7)	0/8 (0)	0/9 (0)	0.415

^1^ Data are presented as mean ± standard deviation, except for the frequency of dams without implantation. Control (*n* = 8), aflatoxins (AF; sum of AFB_1_, AFB_2_, AFG_1_ and AFG_2_: 1.0 mg/kg/day) (*n* = 10), functional yogurt (FY) (*n* = 8), and AF + FY (*n* = 9). No statistically significant differences were observed among groups (*p* > 0.05).

**Table 3:** Fetal and placental morphometric parameters ^1^.

Parameter	Control	AF	FY	AF + FY	*p*-Value
Fetal weight (g)	3.91 ± 0.66 ^a^	3.21 ± 0.28 ^b^	3.90 ± 0.70 ^ab^	3.38 ± 0.36 ^ab^	0.0163
Placental weight (g)	0.63 ± 0.10	0.60 ± 0.14	0.66 ± 0.12	0.61 ± 0.08	0.1326
Body length (cm)	3.62 ± 0.32	3.39 ± 0.19	3.67 ± 0.24	3.42 ± 0.17	0.0395
Tail length (cm)	1.21 ± 0.10	1.12 ± 0.09	1.22 ± 0.10	1.15 ± 0.11	0.1580

^1^ Data are presented as mean ± standard deviation of litter-level means. ^a,b^ In the same row, means with different superscript letters differ significantly (*p* < 0.05). Control (*n* = 8), aflatoxins (AF; sum of AFB_1_, AFB_2_, AFG_1_ and AFG_2_: 1.0 mg/kg/day) (*n* = 9), functional yogurt (FY) (*n* = 8), and AF + FY (*n* = 9). One dam from the AF group was excluded from morphometric analyses because it had no viable fetuses. For body length, ANOVA indicated an overall difference, but Tukey’s post hoc test did not identify significant pairwise differences.

**Table 4:** Occurrence and levels of aflatoxins (AF) in the maternal liver of Wistar rats receiving AF ^1^ alone or in combination with a functional yogurt (FY).

Type of AF	AF Group (*N* = 10)			AF + FY Group (*N* = 8)		
	*n* (%)	Mean (ng/g)	Min.–Max. (ng/g) ^2^	*n* (%)	Mean (ng/g)	Min.–Max. (ng/g) ^2^
M_1_	3 (30)	0.25	0.10–0.53	1 (12.5)	0.10	0.10
P_1_	6 (60)	0.26	0.09–0.89	0	<LOQ	–
Q_1_	0	<LOQ	–	0	<LOQ	–
B_1_	6 (60)	0.84	0.07–3.97	1 (12.5)	0.08	0.08
B_2_	2 (20)	0.53	0.19–0.86	0	<LOQ	–
G_1_	2 (20)	1.22	0.08–2.35	1 (12.5)	0.08	0.08
G_2_	1 (10)	0.08	0.08	0	<LOQ	–

^1^ Sum of AFB_1_, AFB_2_, AFG_1_ and AFG_2_ (1.0 mg/kg/day). ^2^ Minimum and maximum levels calculated using only samples with quantifiable concentrations. *n*: number of samples with concentrations equal to or above the limit of quantification (LOQ: 0.08, 0.09, 0.09, 0.07, 0.15, 0.08 and 0.08 ng/g for aflatoxins M_1_, P_1_, Q_1_, B_1_, B_2_, G_1_ and G_2_, respectively).

**Table 5:** Occurrence and levels of quantifiable aflatoxins (AF) in placental and fetal liver samples from Wistar rats receiving AF ^1^.

Tissue	Type of AF	*n*/*N* (%)	Mean (ng/g)	Min.–Max. (ng/g) ^2^
Placenta	P_1_	4/5 (80)	0.12	0.11–0.13
Placenta	B_1_	2/5 (40)	0.19	0.12–0.25
Fetal liver	M_1_	1/9 (11)	0.12	0.12
Fetal liver	P_1_	4/9 (44)	0.12	0.09–0.18
Fetal liver	B_1_	1/9 (11)	0.81	0.81

^1^ Sum of AFB_1_, AFB_2_, AFG_1_ and AFG_2_ (1.0 mg/kg/day). ^2^ Minimum and maximum levels calculated using only samples with quantifiable concentrations. *n*/*N*: number of samples with concentrations equal to or above the limit of quantification (LOQ: 0.08, 0.09 and 0.09 ng/g for aflatoxins M_1_, P_1_ and B_1_, respectively), relative to the total number of samples evaluated.

## Data Availability

The original contributions presented in this study are included in the article/supplementary material. Further inquiries can be directed to the corresponding authors.

## Supplementary Materials

The following supporting information can be downloaded at https://www.mdpi.com/article/10.3390/toxins18100457/s1: Table S1: Chromatographic conditions used in the analyses for determination of aflatoxins in samples of serum, placenta, and hepatic tissue; Table S2: Mass spectrometry parameters for determination of residual aflatoxins in samples of serum, placenta, and hepatic tissue.
