# Supplementary material for: Effects of Functional Yogurt Supplementation on Aflatoxin-Induced Maternal–Fetal Toxicity in Pregnant Rats
Source: Toxins (Basel). 2026 Oct 20;18(10):457. doi: 10.3390/toxins18100457 (PMC13709716; doi:10.3390/toxins18100457)
Supplement: Supplementary file 1 [file toxins-18-00457-s001.zip › toxins-4586656-supplementary.pdf]

toxins

Supplementary Materials: Effects of Functional Yogurt Supplementation on Aflatoxin-Induced Maternal–Fetal Toxicity in Pregnant Rats

João Victor Batista da Silva, Jéssica Cristina Leme, Giovana Perez Montenegro, Deisy Mara Silva Longo, Marlei Josiele Augusto, Fernando Silva Ramalho, Lucas Gabriel Dionisio Freire, Carlos Humberto Corassin, Fernando Gustavo Tonin, Sher Ali, Carlos Augusto Fernandes de Oliveira and Leandra Náira Zambelli Ramalho

Table S1. Chromatographic conditions used in the analyses for determination of aflatoxins in samples of serum, placenta, and hepatic tissue.

Parameter	Description
Column temperature	40° C
Sample temperature	15° C
Mobile phase	Water (eluent A) and acetonitrile (eluent B), both containing 5.0 mM of ammonium acetate + 0.1% of acetic acid
Gradient elution	The elution gradient began with 98% eluent A for the first 0.5 minutes, followed by a linear increase in eluent B to 30% over the next 4.5 minutes. Eluent B was then raised to 96% over a period of 2.0 minutes and maintained at this level for an additional 0.5 minutes, reaching a total run time of 7.5 minutes. Subsequently, eluent B was decreased to 2% over a 2.0-minute period, and the system was re-equilibrated under initial conditions for another 2.0 minutes
Flow rate	0.6 mL/min
Total chromatographic run time	10.0 min

Table S2. Mass spectrometry parameters for determination of residual aflatoxins in samples of serum, placenta, and hepatic tissue.

Aflatoxin	Retention time (min)	Mass (g/mol)	Molecular ion	Transitions (*m*/*z*)	Cone voltage (V)	Collision energy (V)	LOD range (ng/mL or g)	LOQ range (ng/mL or g)
B_1	5.19	312.3	[M+H]+	312.7 > 284.9 [a]	94	36	0.02-0.03	0.07-0.09
				312.7 > 241.1 [b]		22		
[$^{13}C_{17}$]-B_1	5.19	329.1	[M+H]+	330.3 > 301.5	94	20		
B_2	4.87	314.3	[M+H]+	314.7 > 259.0 [a]	2	28	0.03-0.05	0.15-0.18
				314.7 > 287.0 [b]		26		
G_1	4.85	328.3	[M+H]+	328.9 > 243.0 [a]	2	26	0.03-0.04	0.08-0.10
				328.9 > 199.5 [b]		38		
G_2	4.53	330.3	[M+H]+	330.9 > 245.0 [a]	56	28	0.03-0.06	0.08-0.19
				330.9 > 188.9 [b]		40		
M_1	4.35	328.3	[M+H]+	329.0 > 273.1 [a]	52	24	0.02-0.03	0.06-0.08
				329.0 > 229.0 [b]		38		
Q_1	4.31	328.3	[M+H]+	329.3 > 172.2 [a]	56	30	0.03-0.04	0.09-013
				299.3 > 206.1 [b]		22		
P_1	4.20	298.3	[M+H]+	299.3 > 215.1 [a]	60	34	0.03-0.04	0.09-0.11
				299.3 > 271.0 [b]		24		

[a] transition used for quantification.

[b] transition used for quantification.
